# Preventive Effect of *Lactobacillus fermentum* CQPC03 on Activated Carbon-Induced Constipation in ICR Mice

**DOI:** 10.3390/medicina54050089

**Published:** 2018-11-19

**Authors:** Jing Zhang, Benshou Chen, Baosi Liu, Xianrong Zhou, Jianfei Mu, Qiang Wang, Xin Zhao, Zhennai Yang

**Affiliations:** 1 Beijing Advanced Innovation Center for Food Nutrition and Human Health, Beijing Technology & Business University (BTBU), Beijing 102488, China; zjinger0810@126.com; 2 Environmental and Quality Inspection College, Chongqing Chemical Industry Vocational College, Chongqing 401228, China; cbs7575@sina.com; 3 Chongqing Collaborative Innovation Center for Functional Food, Chongqing University of Education, Chongqing 400067, China; liubs@foods.ac.cn (B.L.); zhouxr@foods.ac.cn (X.Z.); mujianfei@foods.ac.cn (J.M.); wangqiang@foods.ac.cn (Q.W.); 4 College of Food Science, Southwest University, Chongqing 400715, China

**Keywords:** *Lactobacillus fermentum* CQPC03, pickled cabbage, constipation, 16S rDNA, mice

## Abstract

*Background and objectives:* Paocai (pickled cabbage), which is fermented by lactic acid bacteria, is a traditional Chinese food. The microorganisms of Paocai were isolated and identified, and the constipation inhibition effect of one of the isolated *Lactobacillus* was investigated. *Materials and Methods:* The 16S rDNA technology was used for microbial identification. A mouse constipation model was established using activated carbon. After intragastric administration of *Lactobacillus* (10^9^ CFU/mL), the mice were dissected to prepare pathological sections of the small intestine. Serum indicators were detected using kits, and the expression of small intestine-related mRNAs was detected by qPCR assay. *Results:* One strain of *Lactobacillus* was identified and named *Lactobacillus fermentum* CQPC03 (LF-CQPC03). Body weight and activated carbon propulsion rate were all higher in mice intragastrically administered with LF-CQPC03 compared with the control group, while the time to the first black stool in treated mice was lower than that in the control group. Serum assays showed that gastrin (Gas), endothelin (ET), and acetylcholinesterase (AchE) levels were significantly higher in the LF-CQPC03-treated mice than in the control group, while somatostatin (SS) levels were significantly lower than in the control mice. Mouse small intestine tissue showed that c-Kit, stem cell factor (SCF), and glial cell-derived neurotrophic factor (GDNF) mRNA expression levels were significantly higher in the LF-CQPC03 treated mice than in control mice, while transient receptor potential cation channel subfamily V member 1 (TRPV1) and inducible nitric oxide synthase (iNOS) expression levels were significantly lower in the LF-CQPC03 treated mice than in control mice. *Conclusions:* There is a better effect with high-dose LF-CQPC03, compared to the lower dose (LF-CQPC03-L), showing good probiotic potential, as well as development and application value.

## 1. Introduction

Chinese Sichuan Paocai is a traditional fermented food which is made by washing and sealing fresh cabbage into a jar for anaerobic fermentation in brine [1]. Brine plays an important role in the fermentation process, being able to retain microorganisms such as lactic acid bacteria that are beneficial to the fermentation. In brine, the lactic acid bacteria can utilise sugars and nitrogenous substances to proliferate and produce acidic substances, while the lactic acid bacteria can also metabolise flavour components to generate the good flavour [2]. A large number of lactic acid bacteria are found in Paocai, and play a key role in determining Paocai flavour and quality [3]. Studies have shown that the lactic acid bacteria isolated from fermented foods such as Paocai have various benefits for human health, including preventing constipation, colitis, liver damage, and diabetes, and these microorganisms have been used as probiotics [4,5,6,7]. The types of lactic acid bacteria found in Paocai vary with respect to the area, climate, and procedure of Paocai production. Therefore, the microorganisms found in different Paocai products include *Lactobacillus plantarum*, *L. brevis*, *Lactobacillus casei*, and *saccharomycetes*, and *Lactobacillus acidophilus* [8,9,10]. To better isolate and identify beneficial microorganisms, a wider range of isolation and identification procedures is needed to find probiotic strains that can be better applied in industry. East Asian countries consume large amounts of Paocai, and have also carried out related research on different types of it. In addition to research into the effect of the fermentation agent on the lactic acid bacteria in Paocai and the observation of the fermentation of lactic acid bacteria, the biological activity of Paocai lactic acid bacteria itself has also been investigated. The development of functional foods was carried out using the fermentation and self-acting effects of the lactic acid bacteria in Paocai. Our research team also investigated, isolated, and identified the microorganisms in the Paocai produced in Sichuan and Chongqing, China. The strains in this study were also isolated and identified as lactic acid bacteria strains.

Under normal circumstances, probiotic and harmful bacteria in the human intestinal tract are in a state of equilibrium. Probiotics participate in digestion and absorption and remove harmful substances, while harmful bacteria produce harmful substances and destroy intestinal health. Disturbing the equilibrium between probiotic and harmful bacteria can lead to indigestion, intestinal dysfunction, and even malignant diseases such as tumours in severe cases [11,12]. Studies have shown that lactic acid bacteria can inhibit chronic diarrhoea, constipation, bloating, and dyspepsia because lactic acid bacteria can effectively inhibit the growth and reproduction of harmful bacteria through lactic acid metabolism in the gastrointestinal tract, maintain the equilibrium of microbes in the intestine, and maintain normal intestinal function [13]. At the same time, lactic acid bacteria can also activate macrophage phagocytosis, playing an important role in intestinal colonisation. Lactic acid bacteria can also promote cell division and the production of corresponding antibodies, promote cellular immunity, enhance immune responses, and improve disease resistance [14,15]. Constipation refers to a reduction in the number of bowel movements, as well as difficulty in defecation and dry stool caused by poor lifestyle [16]. During constipation, intestinal motility slows, and the harmful bacteria in the intestine may increase greatly. In addition to affecting normal bowel movements, the long-term accumulation of toxic substances in the body will cause other intestinal diseases [17]. Lactic acid bacteria can produce organic acids in the intestine and reduce the pH of the intestinal lumen. At the same time, lactic acid bacteria can regulate the neuromuscular activity of the intestine, enhance intestinal peristalsis, and promote the digestion and absorption by the intestines to help restore and promote intestinal function. In addition, lactic acid bacteria can effectively inhibit the proliferation of harmful bacteria in the intestine, improve the intestinal environment, and soften the faeces to facilitate excretion. A variety of activities resulting from these effects have been observed, including the relief of constipation [18].

In the present study, a mouse constipation model was established using activated carbon to observe the constipation-inhibiting effect of a newly isolated and identified lactic acid bacteria (LF-CQPC03) in Paocai. By detecting the relevant indicators in serum and small intestinal tissue, the recovery of intestinal function by LF-CQPC03 in constipated mice was verified. Additionally, gene expression in the small intestine was determined by qPCR and western blotting (WB) to further elucidate the molecular mechanism of LF-CQPC03 in relieving constipation. The experimental results lay the ground work for better utilisation of strain resources, while the comparison between LF-CQPC03 and commercial strain LB will provide reference data for subsequent industrialisation and application in the pharmaceutical industry.

## 2. Materials and Methods

### 2.1. Isolation and Identification of Lactic Acid Bacteria

For sample preparation, 1 mL of Paocai water sample was collected, and sterile saline was used for 10-fold gradient dilution until reaching 10^−6^ dilution. Subsequently, 100 μL of bacterial solution from the 10^−4^, 10^−5^, and 10^−6^ dilutions was applied onto plates. Colony morphology was observed after 48 h of culture at 37 °C. Then, a single colony was picked for streak culture, which was repeated three times to obtain a pure single colony. The pure strain was inoculated in MRS liquid medium (5 mL) (37 °C, 24 h), and 1 mL of medium containing cultured bacteria was then transferred into a sterile tube and centrifuged (4000 r/min, 10 min). The supernatant was discarded, and the bacterial pellet was subjected to Gram stain microscopy. At the same time, the pure strain was inoculated in MRS broth for culture (37 °C, 24 h). A bacterial genomic DNA extraction kit (Tiangen Biotech (Beijing) Co., Ltd., Beijing, China) was used for DNA extraction, and the extracted DNA was subjected to 16S rDNA amplification. The amplification system contained the following: forward primer (Thermo Fisher Scientific, Inc., Waltham, MA, USA) 27F (5′-AGAGTTTGATCCTG GCTCAG-3′, SEQ ID No.1) 1 μL; reverse primer 1495R (5′-CTACGGCTACCTTGTTACGA-3′, SEQ ID No.2) 1 μL; 2×Taq plus Buffer, 12.5 μL; template DNA, 1 μL; and sterile ddH_2_O to obtain a final volume of 25 μL. Sterile ultrapure water was used instead of template DNA as a negative control. The amplification conditions were 94 °C for 5 min, followed by 29 cycles of 94 °C for 30 s, 55 °C for 30 s, and 72 °C for 1 min, and a final extension at 72 °C for 5 min. Finally, 5 μL of amplified product was subjected to agarose gel electrophoresis (agarose concentration of 1.5%, 110 V, 45 min). The amplified product was subjected to 16S rDNA sequencing for species determination (SimpliAmp Thermal Cycler, Thermo Fisher Scientific, Inc., Waltham, MA, USA) [19].

### 2.2. Animal Experiments

A total of 50 6-week-old, female SPF Kunming mice were purchased from the Experimental Animal Center of Chongqing Medical University and were housed in a standard rearing environment at a temperature of 25 ± 2 °C, 50 ± 5% relative humidity, and a 12 h light/12 h dark cycle. Animal studies were officially started after one week of adaptive feeding. The mice were then randomly divided into 5 groups of 10 mice, including the normal group, the model group, the *Lactobacillus bulgaricus* (LB) gavage group, the low-dose *Lactobacillus fermentum* CQPC03 gavage group (LF-CQPC03-L) and the high-dose *Lactobacillus fermentum* CQPC03 gavage group (LF-CQPC03-H). During the experiment, the mice in the normal and model groups were intragastrically administered 0.2 mL of normal saline per day. The mice in the LB group were intragastrically administered LB (1.0 × 10^9^ CFU/kg), while the mice in the LF-CQPC03-L group and the LF-CQPC03-H group received LF-CQPC03 by gavage (1.0 × 10^8^ CFU/kg and 1.0 × 10^9^ CFU/kg) from the first day in the experiment. From day 7 to 9, mice in all groups except the normal group were intragastrically administered 0.2 mL of 10% activated carbon ice water per day. The body weight of the mice was measured daily during the experiment. On the 9th day, after gavage was completed for the mice in each group, all mice were fasted for 24 h. On the 10th day, all mice were intragastrically administered 0.2 mL of 10% activated carbon ice water, and the mice in each group were then divided into two groups, in which 5 mice were observed for the time of first discharge of black stool after activated carbon ice water gavage, and the remaining 5 mice were observed for the rate of activated carbon propulsion within 30 min after activated carbon ice water gavage. At the same time, the small intestine from the pylorus to the ileocide was collected, and the length of the small intestine and the propelling distance of the activated carbon in the small intestine were measured. The propulsion rate of the small intestine was calculated according to the equation in [20]. The study was conducted in accordance with the Declaration of Helsinki, and the protocol was approved by the Ethics Committee of Chongqing Medical University (No. SYXK 2017-0001).

### 2.3. Determination of Serum Levels of Mice

The plasma was allowed to rest for 1 h, and was then centrifuged at 4500 r/min for 15 min. The serum levels of Gas, ET, SS, and AChE in mice were determined by kits (Nanjing Jiancheng Bioengineering Institute, Nanjing, China).

### 2.4. Pathological Observation of Small Intestine

The 0.5 cm^2^ small intestinal tissues of mice were immobilised in 10% formalin solution for 48 h. The tissues were stained with H&E after dehydration, transparency, wax dipping, embedding, and slicing. The morphological changes were observed under optical microscope (BX43, Olympus, Tokyo, Japan).

### 2.5. Quantitative PCR (qPCR) Assay

Total RNA was extracted from the mouse small intestine using Trizol reagent (Thermo Fisher Scientific, Inc., Waltham, MA, USA) according to the manufacturer’s instructions. Using ultra-micro spectrophotometry, the purity and concentration of the total RNA were determined, and the RNA concentration of each sample was adjusted to 1 μg/μL. Next, 1 μg/μL RNA sample was combined with 1 μL of (oligo) primer dT and 10 μL of sterile ultrapure water and incubated at 65 °C for 5 min. When the reaction was complete, 1 μL of Ribolock RNase Inhibitor (Thermo Fisher Scientific, Inc., Waltham, MA, USA), 2 μL of 100 mM dNTP mix, 4 μL of 5× Reaction buffer, and 1 μL of Revert Aid M-mu/v RT were added to the reaction system to synthesise the cDNA at 42 °C for 60 min and 70 °C for 5 min. The target gene (Table 1, Thermo Fisher Scientific, Inc., Waltham, MA, USA) was then reverse transcribed and amplified under the following conditions: denaturation at 95 °C for 15 min, followed by 40 cycles of annealing at 60 °C for 1 h, and an extension at 95 °C for 15 min. DAPDH was used as a housekeeping gene, and the relative expression of the target gene was calculated as 2^−ΔΔ*C*t^ method [21].

### 2.6. Statistical Analysis

Differences between the mean values for each group mice were analyzed by a one-way ANOVA with Duncan’s multiple range tests using SAS v9.1 statistical software package (SAS Institute Inc., Cary, NC, USA).

## 3. Results

### 3.1. Isolation and Identification of Microorganisms

**Figure 1:**
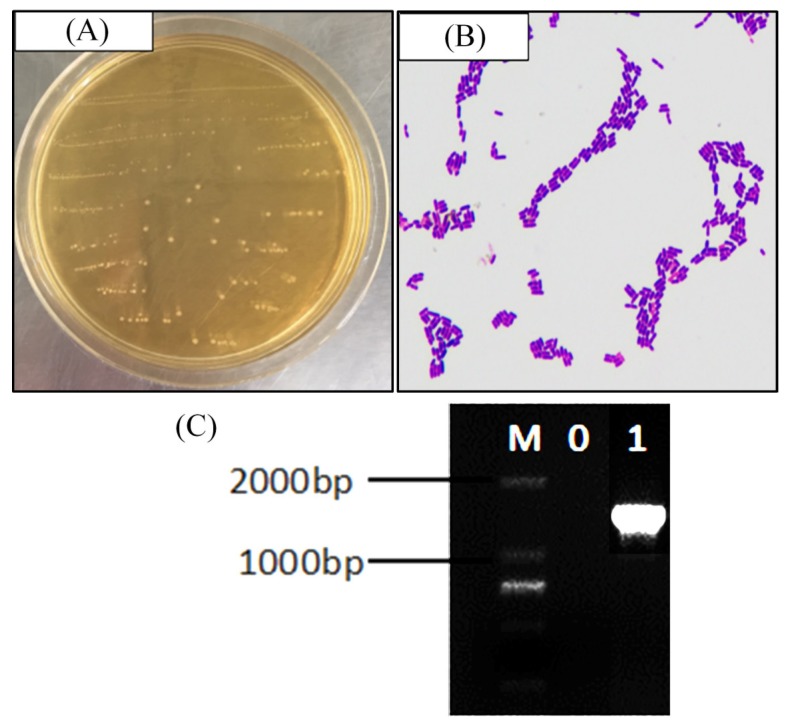
Gram staining result (**A**), colony morphology (**B**) and 16S rDNA agarose gel electrophoresis of PCR amplified product (**C**) of *Lactobacillus fermentum* CQPC03. M: 2000bp DNA Ladder; 0: negative control group; 1: *Lactobacillus fermentum* CQPC03.

Visually, the strain colonies were mostly round white or milky white, with a clean border at the colony edge and a moist and smooth colony surface (Figure 1A). Gram staining was used to preliminarily identify strains as lactic acid bacteria. Under a microscope, strains had long and short rods, with no budding reproduction (Figure 1B). Gel electrophoresis revealed no bands in the negative control group lane, and a band of approximately 1500 bp in the test strain lane (Figure 1C), indicating that the PCR was not contaminated and that 16S rDNA sequencing could proceed. The sequencing results showed that the test strain showed 99% homology with known fermented lactic acid bacteria in GenBank (GenBank accession number: NC_004567.2). Being a newly-discovered fermented lactic acid bacteria, the strain was named *Lactobacillus fermentum* CQPC03, and it was preserved in China General Microbiological Culture Collection Center (CGMCC, Beijing, China), the strain preservation number was CGMCC No. 14492.

### 3.2. Changes in Mouse Body Weight

**Figure 2:**
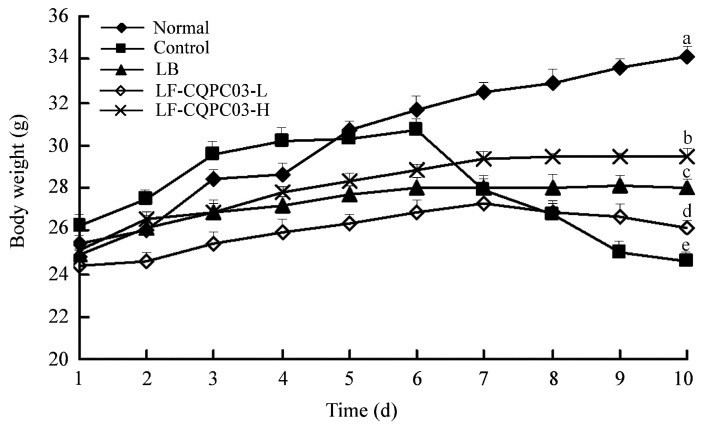
The body weight of mice in the experment. Values presented are the mean ± standard deviation. ^a–e^ According to Duncan’s multiple-range test, different letters indicate significant differences (*p* < 0.05) between each other. LB: *Lactobacillus bulgaricus* (1.0 × 10^9^ CFU/kg b.w.); LF-CQPC03-L: *Lactobacillus fermentum* CQPC03 (1.0 × 10^8^ CFU/kg b.w.); LF-CQPC03-H: *Lactobacillus fermentum* CQPC03 (1.0 × 10^9^ CFU/kg b.w.).

As shown in Figure 2, the mice in each group showed normal growth for the first six days; after the administration of activated carbon water from the seventh day, the weight of the mice decreased in each group except for the normal group. The weight loss of the mice in the control group was the greatest, and the weight loss of the mice in the LB- and LF-CQPC03-H groups was slowest. On the last day, the weight of the mice in the LF-CQPC03-H group was only lower than that in the normal group. Accordingly, the body weight of the mice was affected by constipation, and LF-CQPC03 alleviated this decline in body weight to a certain degree. A higher concentration of LF-CQPC03 showed a better inhibitory effect on constipation-induced weight loss.

### 3.3. The Defecation of Mice

As shown in Table 2, from the first to the seventh day, there was no significant (*p* > 0.05) difference in fecal status (stool weight, particle count of stool, and water content of stool) of mice in each group. From the eighth day, the stool weight, particle count of stool and water content of stool of mice feces began to decline due to the action of active carbon. LF-CQPC010 and LB could inhibit these changes, and the LP-CQPC03-H showed the better effects than LP-CQPC03-L and LB.

### 3.4. The Time to the First Black Stool Discharge for the Mice

**Figure 3:**
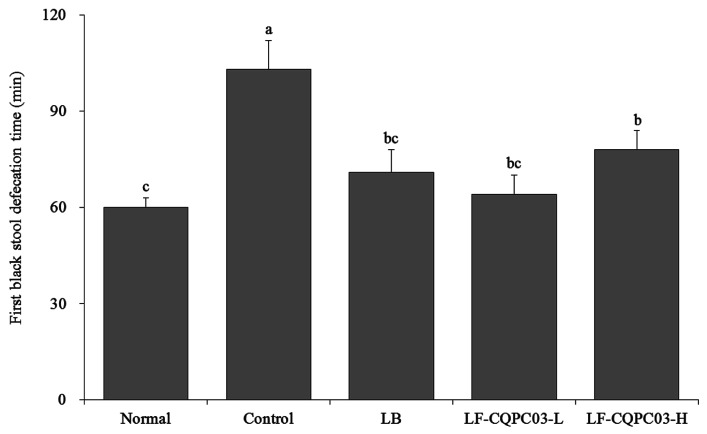
First black stool defecation time of the mice. Values presented are the mean ± standard deviation. ^a–c^ According to Duncan’s multiple-range test, different letters indicate significant differences (*p* < 0.05) between each other. LB: *Lactobacillus bulgaricus* (1.0 × 10^9^ CFU/kg b.w.); LF-CQPC03-L: *Lactobacillus fermentum* CQPC03 (1.0 × 10^8^ CFU/kg b.w.); LF-CQPC03-H: *Lactobacillus fermentum* CQPC03 (1.0 × 10^9^ CFU/kg b.w.).

As shown in Figure 3, the mice with the shortest discharge time for the first black stool were those in the normal group, and the mice with the longest discharge time for the first black stool were those in the control group, indicating that intestinal peristalsis was weakest, and that defecation was the most difficult in the control mice. The time to the first black stool discharge for the mice after LF-CQPC03 gavage was significantly reduced compared with the control group. Thus, LF-CQPC03 significantly improved intestinal peristalsis in mice, thereby effectively promoting defecation.

### 3.5. Activated Carbon Propulsion Rate in the Small Intestine of the Mice

As shown in Table 3, the propulsion rate of the activated carbon water was lowest in the small intestine of the control mice with constipation induction, and was higher in the mice treated with LF-CQPC03-H compared with all groups except for the normal group. At the same time, no significant difference in the propulsion rate of the activated carbon water in the small intestine was observed between mice treated with LB and LB-LFQC03-L. In summary, LF-CQPC03 can alleviate decreased intestinal peristalsis due to constipation in a dose-dependent manner.

### 3.6. Serum ET, SS, AChE and Gas Levels in the Mice

As shown in Table 4, the normal group showed the lowest serum SS levels and highest serum SS, AChE, and ET levels, while the control group showed the opposite trend, with the highest SS levels and the lowest SS, AChE, and ET levels. ET, AChE, and Gas levels in the serum of the LF-CQPC03-H gavage mice were only lower than those in the normal group, and the SS level was also only higher than that in the normal group. The levels of ET, AChE and Gas in LF-CQPC03-L- and LB-treated mice were also higher than those in the control group, and SS levels were lower than those in the control group.

### 3.7. Pathological Observation of Small Intestine Tissue

**Figure 4:**
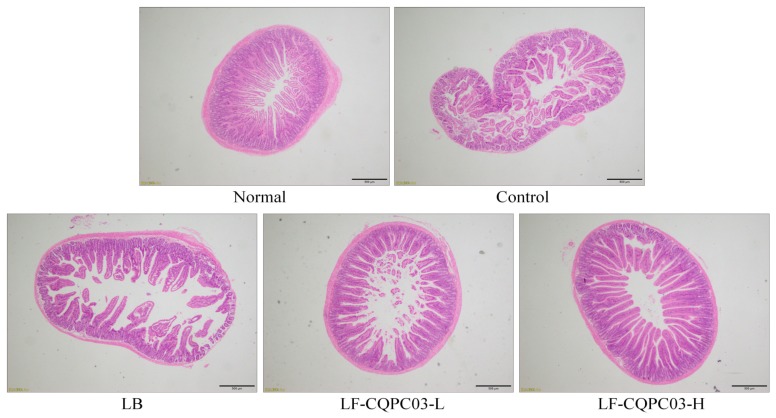
Morphological observation of small-intestine tissue in mice with activated carbon-induced constipation. LB: *Lactobacillus bulgaricus* (1.0 × 10^9^ CFU/kg b.w.); LF-CQPC03-L: *Lactobacillus fermentum* CQPC03 (1.0 × 10^8^ CFU/kg b.w.); LF-CQPC03-H: *Lactobacillus fermentum* CQPC03 (1.0 × 10^9^ CFU/kg b.w.).

As shown in Figure 4, the small intestine of mice in the normal group displayed an intact structure, with a uniform and smooth intestinal wall and neatly arranged villi. After the induction of constipation using activated carbon, the intestinal wall of the mice in the control group became rougher, and fractures and shrinkage were visible in a large number of villi. LB and LF-CQPC03 gavage both reduced the breakage and shrinkage of the villi in treated mice and preserved the smooth appearance of the small intestine wall. LF-CQPC03-H showed the greatest efficacy at minimising the damage to the small intestine, with the appearance of the villi and small intestine wall closely mirroring those in mice from the normal group.

### 3.8. c-Kit mRNA Expression in the Small Intestine

**Figure 5:**
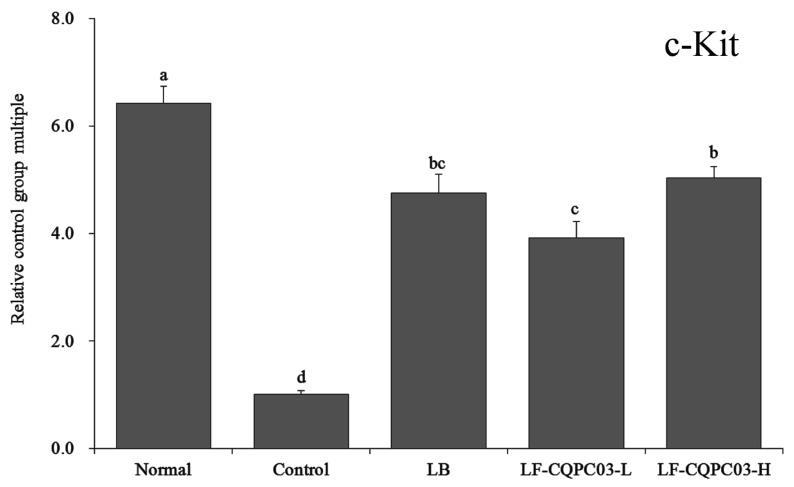
mRNA expression level of c-Kit in the small-intestine tissue of mice. Values presented are the mean ± standard deviation. ^a–d^ According to Duncan’s multiple-range test, different letters indicate significant differences (*p* < 0.05) between each other. LB: *Lactobacillus bulgaricus* (1.0 × 10^9^ CFU/kg b.w.); LF-CQPC03-L: *Lactobacillus fermentum* CQPC03 (1.0 × 10^8^ CFU/kg b.w.); LF-CQPC03-H: *Lactobacillus fermentum* CQPC03 (1.0 × 10^9^ CFU/kg b.w.).

As shown in Figure 5, c-Kit mRNA expression was the weakest in the small intestine of the control group, and the relative expression of c-Kit in the small intestine of the normal group was the highest, being 6.42 times of that of the control group. c-Kit expression in the small intestine of constipated mice was up-regulated by LB and LF-CQPC03 treatment. The expression levels of c-Kit in the small intestine of the LB, LF-CQPC03-L, and LF-CQPC03-H mice were 4.75, 3.92, and 5.04 times that in the control mice, respectively.

### 3.9. SCF mRNA Expression in the Small Intestine

**Figure 6:**
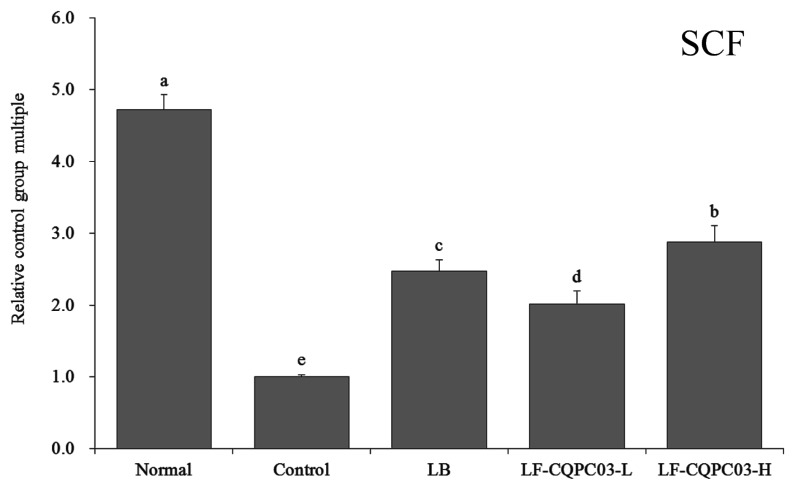
mRNA expression level of SCF in the small-intestine tissue of mice. Values presented are the mean ± standard deviation. ^a–e^ According to Duncan’s multiple-range test, different letters indicate significant differences (*p* < 0.05) between each other. LB: *Lactobacillus bulgaricus* (1.0 × 10^9^ CFU/kg b.w.); LF-CQPC03-L: *Lactobacillus fermentum* CQPC03 (1.0 × 10^8^ CFU/kg b.w.); LF-CQPC03-H: *Lactobacillus fermentum* CQPC03 (1.0 × 10^9^ CFU/kg b.w.).

As shown in Figure 6, the expression of stem cell factor (SCF) was the weakest in the small intestine of the control group; SCF expression levels in the small intestine of the normal, the LB, the LF-CQPC03-L, and the LF-CQPC03-H groups were 4.72, 2.47, 2.01, and 2.88 times that in the control group, respectively. The expression of SCF in the small intestine of the LF-CQPC03-H group was significantly higher than that in the LB and LF-CQPC03-L groups.

### 3.10. GDNF mRNA Expression in the Small Intestine

**Figure 7:**
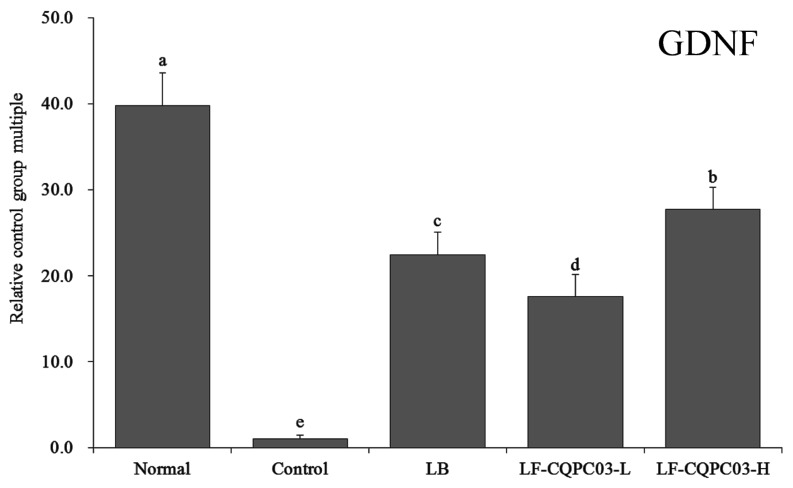
mRNA expression level of GDNF in the small-intestine tissue of mice. Values presented are the mean ± standard deviation. ^a–e^ According to Duncan’s multiple-range test, different letters indicate significant differences (*p* < 0.05) between each other. LB: *Lactobacillus bulgaricus* (1.0 × 10^9^ CFU/kg b.w.); LF-CQPC03-L: *Lactobacillus fermentum* CQPC03 (1.0 × 10^8^ CFU/kg b.w.); LF-CQPC03-H: *Lactobacillus fermentum* CQPC03 (1.0 × 10^9^ CFU/kg b.w.).

The expression levels of glial cell-derived neurotrophic factor (GDNF) mRNA in the small intestine of each group are shown in Figure 7. GDNF expression was strongest in the small intestine of the normal group (39.73 ± 3.87 times that in control mice); after the induction of constipation, GDNF expression decreased in the small intestine of the control group; LB, LF-CQPC03-L, and LF-CQPC03-L all up-regulated GDNF expression in the small intestine of mice with constipation, and the effect of LF-CQPC03-H (27.69 ± 2.61 times that in control mice) on GDNF up-regulation was stronger than that of LB (22.43 ± 2.66 times that in control mice) and LF-CQPC03-L (17.61 ± 2.52 times that in control mice).

### 3.11. TRPV1 mRNA Expression in the Small Intestine

**Figure 8:**
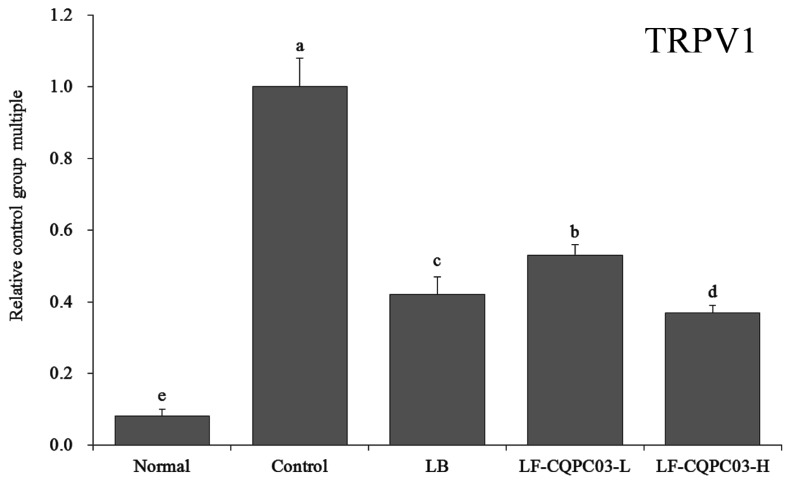
mRNA expression level of TRPV1 in the small-intestine tissue of mice. Values presented are the mean ± standard deviation. ^a–e^ According to Duncan’s multiple-range test, different letters indicate significant differences (*p* < 0.05) between each other. LB: *Lactobacillus bulgaricus* (1.0 × 10^9^ CFU/kg b.w.); LF-CQPC03-L: *Lactobacillus fermentum* CQPC03 (1.0 × 10^8^ CFU/kg b.w.); LF-CQPC03-H: *Lactobacillus fermentum* CQPC03 (1.0 × 10^9^ CFU/kg b.w.).

As shown in Figure 8, the level of transient receptor potential cation channel subfamily V member 1 (TRPV1) expression in mice from each group were significantly higher than that in the normal group (0.08 ± 0.02 times that in control mice). The expression of TRPV1 in the small intestine of the mice in the LF-CQPC03-H group (0.37 ± 0.02 times that in control mice) was closest to that in the normal group, which was significantly lower than in the mice from the LB (0.42 ± 0.05 times that in control mice) and LF-CQPC03-L groups (0.53 ± 0.03 times that in control mice).

### 3.12. iNOS mRNA Expression in the Small Intestine

**Figure 9:**
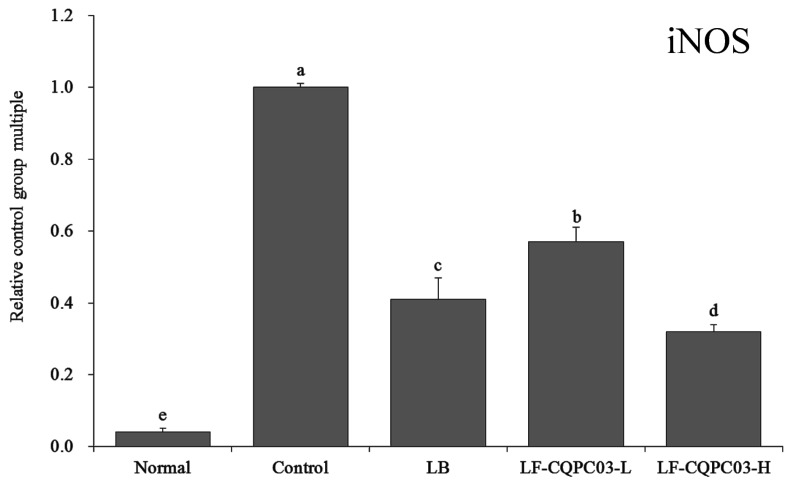
mRNA expression level of iNOS in the small-intestine tissue of mice. Values presented are the mean ± standard deviation. ^a–e^ According to Duncan’s multiple-range test, different letters indicate significant differences (*p* < 0.05) between each other. LB: *Lactobacillus bulgaricus* (1.0 × 10^9^ CFU/kg b.w.); LF-CQPC03-L: *Lactobacillus fermentum* CQPC03 (1.0 × 10^8^ CFU/kg b.w.); LF-CQPC03-H: *Lactobacillus fermentum* CQPC03 (1.0 × 10^9^ CFU/kg b.w.).

As shown in Figure 9, the expression of inducible nitric oxide synthase (iNOS) was the lowest in the small intestine of the normal group (0.04 ± 0.01 times that of the control group), and iNOS expression in the control group was opposite that in the normal group. After LF-CQPC03-H treatment, the iNOS expression in the mice with constipation was closest that of the normal group (0.32 ± 0.02 times that in the control group), and was also significantly lower than that in the mice with constipation after LB (0.41 ± 0.06 times of that of the control mice) and LF-CQPC03-L (0.57 ± 0.04 times of that of the control mice) treatment.

## 4. Discussion

Constipation can cause a decline in quality of life. Long-term constipation not only affects normal life, but also causes other intestinal diseases and even malignant diseases [4]. Constipation can also cause symptoms such as bloating and loss of appetite, which can lead to a decline in body constitution and weight loss. Animal studies have also shown that constipation can cause weight loss, less defecation, and dry feces in mice [4,5]. In the present study, mice were also found to exhibit weight loss, less defecation, and dry feces after the induction of constipation. LF-CQPC03 inhibited the decline of body weight, less defecation, dry feces, and relieved constipation. Constipation can cause an increase in the amount of harmful microorganisms in the intestine, thereby destroying the tissue of the small intestine and causing damage to the intestinal wall and the intestinal villi, such that intestinal peristaltic function is also greatly affected, which can cause or aggravate constipation. Therefore, evaluating the integrity of the tissue in the small intestine is also a means of detecting constipation, including observation of the integrity of the villi in the intestinal wall [17]. The present study also observed pathological changes in small intestine tissue caused by constipation, and LF-CQPC03 effectively protected the integrity of the small intestine tissue and minimised the damage to the intestinal wall and villi caused by constipation. In addition, the most obvious symptom of constipation is difficulty in defecation. Therefore, in the present study, the time for animals to discharge the first black stool after receiving activated carbon is also an important indicator for evaluating constipation. Because constipation can slow intestinal peristalsis and cause activated carbon to be retained in the intestinal tract, the time to discharge black stools is delayed [17]. In the present study, the time to discharge black stool in the constipation mice was longer than that of the normal mice, and was significantly reduced by LF-CQPC03, indicating the relief of constipation.

Endothelin (ET) plays an important role in cardiovascular and intestinal function, being able to relax blood vessels and thus maintain normal blood vessel and nerve function in the intestine. At the same time, the balance between ET and NO is important to avoid vascular endothelial damage and haemodynamic disorder and enable normal intestinal activity [22,23]. Somatostatin (SS) also has a direct stimulatory effect on constipation. A large increase in SS levels will lead to the decreased release of gastrointestinal hormones, a significant decrease in the gastric emptying rate, a slowing of smooth muscle contraction, and disordered of intestinal activity, leading to constipation [4,23]. Acetylcholinesterase (AchE) is a neurotransmitter that plays an important role in intestinal motility. AchE binding to its receptor promotes gastrointestinal motility and enhances bowel movements. Increased AchE levels can significantly reduce the impact of constipation on the body [24]. Gastrin (Gas) is also an important gastrointestinal hormone that has many functions in relieving constipation and improving intestinal vitality, including promoting the secretion of gastric juice, increasing intestinal peristalsis, accelerating gastric emptying, and promoting the relaxation of the pyloric sphincter [25], in agreement with the animal studies reported here. Constipation resulted in decreased ET, AchE, and Gas levels, and enhanced SS levels in mice; LF-CQPC03 significantly inhibited these changes, restoring the the above indicators in mice with induced constipation to levels near those of normal mice, and the effect was better than the commonly-used commercial strain LB.

Cajal cells (ICCs) are mesenchymal cells associated with intestinal health. Abnormalities in ICC content and structure in the intestine can slow down intestinal peristalsis, causing intestinal dysfunction and constipation [26]. Some specific markers of ICCs are known, of which c-Kit is among the most important, and can significantly affect ICC function. Disorder of a large number of ICCs will cause intestinal diseases, while SCF is a ligand for c-Kit receptors, and the recovery of the intestinal tract can also be promoted by enhancing the level of SCF [27]. Clinical studies have also confirmed that ICC density is decreased in the small intestine of patients with constipation, and the main cause of the decrease in ICC density is the decrease in c-Kit gene expression in the intestine [28]. The present study also showed that LF-CQPC03 effectively alleviated the effects of constipation on c-Kit and SCF expression in the small intestine of mice and preserved near-normal levels of c-Kit and SCF expression.

TRPV1 can promote the release of neurotransmitters, causing intestinal dyskinesia and difficulty in defecation. Clinical studies have reported increased TRPV1 expression in constipation patients with intestinal damage [29]. GDNF is also an important factor associated with constipation. GDNF can exert its neuromodulatory effects to repair the damaged intestinal tract, increase intestinal movement, and relieve constipation [30]. In the present study, LF-CQPC03 also inhibited constipation by regulating TRPV1 and GDNF expression levels. Abnormal increases in NO content in the intestine can also cause intestinal dyskinesia [31]. NOS is an important factor that regulates NO in the body. Increased iNOS content will cause an increase in NO content, leading to constipation [32]. The present study also found that LF-CQPC03 relieved constipation by down-regulating iNOS expression.

## 5. Conclusions

This study investigated the inhibitory effect of a newly-isolated lactic acid bacteria from Paocai on constipation. LF-CQPC03 improved the related indicators in the serum and small intestinal tissues of constipated mice, and significantly alleviated the effects of constipation on the body. Pathological observation and molecular biological experiments further confirmed the intestinal-protective effect of LF-CQPC03, and its effect was significantly better than that of an identical dose of the commonly-used commercial strain LB. The effect of LF-CQPC03 was also positively correlated with the dose. Therefore, LF-CQPC03 has a good experimental constipation-prevention effect, and shows probiotic potential. LF-CQPC03 is a high-quality microbial resource that is expected to be applied in the medical and food industries.

## Figures and Tables

**Table 1:** Sequences of primers used in this study.

Gene Name	Sequence
c-Kit	Forward: 5′-AGA CCG AAC GCA ACT T-3′
	Reverse: 5′-GGT GCC ATC CAC TTC A-3′
SCF	Forward: 5′-AAA CTG GTG GCG AAT C-3′
	Reverse: 5′-CAC GGG TAG CAA GAA C-3′
GDNF	Forward: 5′-TTT TAT TCA AGC CAC CAT C-3′
	Reverse: 5′-AGC CCA AAC CCA AGT CA-3′
TRPV1	Forward: 5′-AGC GAG TTC AAA GAC CCA GA-3′
	Reverse: 5′-TTC TCC ACC AAG AGG GTC AC-3′
iNOS	Forward: 5′-AGA GAG ATC GGG TTC ACA-3′
	Reverse: 5′-CAC AGA ACT GAG GGT ACA-3′
GAPDH	Forward: 5′-AGG TCG GTG TGA ACG GAT TTG-3′
	Reverse: 5′-GGG GTC GTT GAT GGC AAC A-3′

SCF: stem cell factor; GDNF: glial cell-derived neurotrophic factor; TRPV1: transient receptor potential cation channel subfamily V member 1; NOS: nitric oxide synthase; iNOS: inducible nitric oxide synthase.

**Table 2:** Stool status of mice with activated carbon-induced constipation.

Groups	Normal	Control	LB	LP-CQPC03-L	LP-CQPC03-H
1–7 d (lactic acid bacteria administration period but not induction of constipation)					
Stool weight (g)	1.10 ± 0.03 ^a^	1.08 ± 0.05 ^a^	1.11 ± 0.05 ^a^	1.10 ± 0.04 ^a^	1.13 ± 0.05 ^a^
Particle count of stool	43 ± 3 ^A^	44 ± 3 ^A^	45 ± 3 ^A^	45 ± 2 ^A^	44 ± 5 ^A^
Water content of stool (%)	52 ± 5 ^a^	51 ± 5 ^a^	50 ± 4 ^a^	51 ± 5 ^a^	51 ± 4 ^a^
8–10 d (lactic acid bacteria administration period, induction of constipation)					
Stool weight (g)	1.16 ± 0.06 *^a^*	0.44 ± 0.07 *^d^*	0.71 ± 0.06 *^c^*	0.76 ± 0.05 *^c^*	0.99 ± 0.05 *^b^*
Particle count of stool	53 ± 2 *^A^*	21 ± 6 *^D^*	38 ± 5 *^C^*	40 ± 3 *^C^*	47 ± 2 *^B^*
Water content of stool (%)	53 ± 4 *^a^*	17 ± 5 *^d^*	33 ± 5 *^c^*	36 ± 4 *^c^*	46 ± 3 *^b^*

Values presented are the mean ± standard deviation. ^a, A, a, *a*–*d*, *A*–*D*, *a*–*d*^ According to Duncan’s multiple-range test, different letters indicate significant differences (*p* < 0.05) between each other, while the same letters indicate that there is no significant difference (*p* > 0.05) in the same row. LB: *Lactobacillus bulgaricus* (1.0 × 10^9^ CFU/kg b.w.); LF-CQPC03-L: *Lactobacillus fermentum* CQPC03 (1.0 × 10^8^ CFU/kg b.w.); LF-CQPC03-H: *Lactobacillus fermentum* CQPC03 (1.0 × 10^9^ CFU/kg b.w.).

**Table 3:** Gastrointestinal (GI) transit in mice with activated carbon-induced constipation.

Groups	Length of Small Intestine (cm)	Length of GI Transit (cm)	Activated Carbon Propulsive Rate (%)
Normal	52.3 ± 4.2 ^ab^	52.3 ± 4.2 ^A^	100.00 ± 7.36 ^a^
Control	52.8 ± 5.1 ^ab^	23.0 ± 3.7 ^D^	43.26 ± 7.03 ^d^
LB	49.4 ± 7.4 ^ab^	31.4 ± 5.6 ^C^	68.64 ± 8.32 ^c^
LF-CQPC03-L	47.8 ± 2.7 ^b^	29.6 ± 3.6 ^C^	62.20 ± 5.15 ^c^
LF-CQPC03-H	56.0 ± 1.7 ^a^	44.4 ± 1.7 ^D^	76.05 ± 3.22 ^b^

Values presented are the mean ± standard deviation. ^a–b, A–D, a–d^ According to Duncan’s multiple-range test, different letters indicate significant differences (*p* < 0.05) between each other, while the same letters indicate that there is no significant difference (*p* > 0.05) in the same column. LB: *Lactobacillus bulgaricus* (1.0 × 10^9^ CFU/kg b.w.); LF-CQPC03-L: *Lactobacillus fermentum* CQPC03 (1.0 × 10^8^ CFU/kg b.w.); LF-CQPC03-H: *Lactobacillus fermentum* CQPC03 (1.0 × 10^9^ CFU/kg b.w.).

**Table 4:** GAS, ET, SS, AchE, SP, and VIP serum levels in mice with activated carbon-induced constipation.

Levels (pg/mL)	Normal	Control	LB	LF-CQPC03-L	LF-CQPC03-H
ET	18.2 ± 0.9 ^a^	5.1 ± 0.5 ^e^	13.7 ± 0.5 ^c^	12.1 ± 0.2 ^d^	16.4 ± 0.1 ^b^
SS	35.4 ± 0.7 ^D^	74.8 ± 2.9 ^A^	41.8 ± 1.5 ^C^	50.9 ± 0.6 ^B^	40.6 ± 1.3 ^C^
AchE	28.3 ± 0.1 ^a^	8.4 ± 0.2 ^e^	20.1 ± 0.5 ^c^	18.3 ± 0.8 ^d^	23.6 ± 0.1 ^b^
Gas	79.5 ± 0.8 *^a^*	36.1 ± 0.4 *^e^*	56.2 ± 0.1 *^c^*	48.2 ± 0.2 *^d^*	64.5 ± 0.7 *^b^*

Values presented are the mean ± standard deviation. ^a–e, A–D, a–e, *a*–*e*^ According to Duncan’s multiple-range test, different letters indicate significant differences (*p* < 0.05) between each other, while the same letters indicate that there is no significant difference (*p* > 0.05) in the same row. LB: *Lactobacillus bulgaricus* (1.0 × 10^9^ CFU/kg b.w.); LF-CQPC03-L: *Lactobacillus fermentum* CQPC03 (1.0 × 10^8^ CFU/kg b.w.); LF-CQPC03-H: *Lactobacillus fermentum* CQPC03 (1.0 × 10^9^ CFU/kg b.w.). ET, endothelin; SS, somatostatin; AchE, acetylcholinesterase; Gas, gastrin.
